# Investigating the impact of preselection on subsequent single-step genomic BLUP evaluation of preselected animals

**DOI:** 10.1186/s12711-020-00562-6

**Published:** 2020-07-29

**Authors:** Ibrahim Jibrila, Jan ten Napel, Jeremie Vandenplas, Roel F. Veerkamp, Mario P. L. Calus

**Affiliations:** Wageningen University and Research Animal Breeding and Genomics, Droevendaalsesteeg 1, Wageningen, 6708PB The Netherlands

## Abstract

**Background:**

Preselection of candidates, hereafter referred to as preselection, is a common practice in breeding programs. Preselection can cause bias and accuracy loss in subsequent pedigree-based best linear unbiased prediction (PBLUP). However, the impact of preselection on subsequent single-step genomic BLUP (ssGBLUP) is not completely clear yet. Therefore, in this study, we investigated, across different heritabilities, the impact of intensity and type of preselection on subsequent ssGBLUP evaluation of preselected animals.

**Methods:**

We simulated a nucleus of a breeding programme, in which a recent population of 15 generations was produced with PBLUP-based selection. In generation 15 of this recent population, the parents of the next generation were preselected using several preselection scenarios. These scenarios were combinations of three intensities of preselection (no, high or very high preselection) and three types of preselection (genomic, parental average or random), across three heritabilities (0.5, 0.3 or 0.1). Following each preselection scenario, a subsequent evaluation was performed using ssGBLUP by excluding all the information from the preculled animals, and these genetic evaluations were compared in terms of accuracy and bias for the preselected animals, and in terms of realized genetic gain.

**Results:**

Type of preselection affected selection accuracy at both preselection and subsequent evaluation stages. While preselection accuracy decreased, accuracy in the subsequent ssGBLUP evaluation increased, from genomic to parent average to random preselection scenarios. Bias was always negligible. Genetic gain decreased from genomic to parent average to random preselection scenarios. Genetic gain also decreased with increasing intensity of preselection, but only by a maximum of 0.1 additive genetic standard deviation from no to very high genomic preselection scenarios.

**Conclusions:**

Using ssGBLUP in subsequent evaluations prevents preselection bias, irrespective of intensity and type of preselection, and heritability. With GPS, in addition to reducing the phenotyping effort considerably, the use of ssGBLUP in subsequent evaluations realizes only a slightly lower genetic gain than that realized without preselection. This is especially the case for traits that are expensive to measure (e.g. feed intake of individual broiler chickens), and traits for which phenotypes can only be measured at advanced stages of life (e.g. litter size in pigs).

## Background

Selection of the parents of the next generation usually takes place in two or more stages (e.g. [1–3].), and the term ‘preselection’ is used to refer to the early stages of selection (e.g. [3–5]). Preselection is a common practice in the nuclei of breeding programs, where only a few hundred to a few thousand replacement animals are required per generation. In order to have a large pool of animals to select from, many more young animals are produced than the numbers required for producing the next generation. Preselection is done for different reasons for different traits. For traits that are difficult or expensive to measure (e.g. feed intake of individual broiler chickens), preselection is used to reduce phenotyping costs. For traits for which phenotypes can be measured only at advanced stages of life (e.g. litter size in pigs), preselection is used to reduce the cost of raising the animals until phenotyping. Traditionally, preselection has mostly been based on correlated trait(s) that can be measured easily and cheaply early in life (e.g. [1, 3, 6–8]). In the genomic era, preselection is often based on genomic estimated breeding values (GEBV) of young selection candidates, and in the literature this type of preselection is called genomic or genotypic preselection (GPS; e.g. [4, 5, 9]).

Before the introduction of genomic prediction [10], models for the genetic evaluation of animals were based on phenotypic and pedigree data. These models are generally easy to implement and run fast, but their limitation is that they provide low accuracies for animals without own phenotype (e.g. [11, 12]). With the progress in DNA technology, large-scale genotyping of animals became affordable and genomic information can now be included in genetic evaluations of animals, e.g. by using multi-step genomic evaluation models, where genomic and pedigree information are used in two separate steps [13]. Generally, multi-step genomic evaluation models estimate breeding values more accurately than pedigree-based models, but have the disadvantage of estimating breeding values for genotyped animals only (e.g. [11, 12].). Because the required reference population (animals with genotypes and phenotypes) for multi-step models are usually already selected, the breeding values obtained are biased (e.g. [11, 12].). In 2010, single-step genomic evaluation models were introduced as improvements over both pedigree-based and multi-step genomic models [14, 15]. Single-step models combine all available pedigree, genomic and phenotypic information and provide GEBV for all the animals regardless of whether the animals have phenotypes and/or genotypes. It has been shown that single-step models produce more accurate and less biased breeding values than pedigree-based and multi-step genomic models, even in the presence of selective genotyping and phenotyping (e.g. [16, 17]).

Preselection is known to result in a positive average Mendelian sampling (MS) term for the selected animals (e.g. [4, 18, 19]). Selection candidates that have a positive average MS term represent a violation of one of the assumptions of genetic evaluation models (i.e. that the expectation of the average MS of the observed offspring is zero). This has been reported to result in biased and less accurate estimated breeding values (EBV) in subsequent evaluations that are done using pedigree-based best linear unbiased prediction (PBLUP, e.g. [4, 18–22]). It is also known that when all the information on which preselection is based is included in the subsequent PBLUP evaluations, the impact of the violation of this assumption is usually alleviated, e.g. [20–22].

Single-step genomic BLUP (ssGBLUP) has been reported to handle GPS better than PBLUP. For example, Masuda et al. [5] reported lower genetic trends in milk, fat, and protein yields in genomically-preselected US Holsteins when the subsequent evaluations were performed with PBLUP than with ssGBLUP. These authors ([5]) used these differences in genetic trends between PBLUP and ssGBLUP as an evidence of preselection bias in PBLUP evaluations following GPS. Although Aguilar et al. [14] hypothesised that ssGBLUP could completely prevent preselection bias, to date, there is no study in the literature that compared results using the same data with and without preselection to investigate this hypothesis. The study of Masuda et al. [5] evaluated preselection bias in subsequent PBLUP and ssGBLUP evaluations, but did not include a scenario based on the complete data (without preselection), with which the other scenarios could be compared. Furthermore, the benefit of including the genotypes of the selection candidates discarded at the preselection stage–hereafter referred to as preculled animals–in subsequent ssGBLUP evaluations is still not clear. On the one hand, Shabalina et al. [23] concluded that including the genotypes of preculled animals in subsequent ssGBLUP evaluations improves accuracy in situations where (some of the) parents of the genotyped selection candidates are not genotyped. On the other hand, Koivula et al. [24] reported larger biases and losses in reliability in subsequent ssGBLUP evaluations when genotypes of preculled animals were included and most of the parents of the selection candidates were genotyped. Thus, our aim was to investigate the impact of preselection on subsequent evaluations of preselected animals, using ssGBLUP with all the information from the preculled animals excluded.

## Methods

### Data simulation

To achieve our aim, we simulated a nucleus of a breeding program with inputs from the international breeding companies that operate in the Netherlands, using QMSim [25]. The QMSim parameter file, with all the details of the simulation, are in Additional file 1. For each animal in the breeding program, a genome of 30 chromosomes each 100 cM long was simulated. Sixty thousand single nucleotide polymorphisms (SNPs) and 3000 quantitative trait loci (QTL) were evenly distributed across the entire genome, and the QTL effects were randomly drawn from a gamma distribution with a shape parameter of 0.4. The simulation started with a historical population, to establish mutation-drift equilibrium and linkage disequilibrium among markers and QTL. The historical population had 3000 generations of random mating, starting with 2500 female and 2500 male animals (both sexes were equally represented throughout the simulation). The size of the historical population decreased linearly until it reached 50 animals at generation 2997, and then increased and reached 5000 animals again at generation 3000. The founder population, which comprised 100 males and 1000 females, was randomly selected from the 3000th historical generation. Then, from this founder population, 15 (recent) generations of artificial selection were simulated. In each generation, 100 males and 1000 females were selected and mated to produce the next generation of 16,000 animals. Within sex, all selected parents contributed equally to the next generation. Selection was based on EBV, and the mating design aimed at minimising inbreeding by using minimum co-ancestry matings as described in [26], which minimize the average relationship among all sires and dams, and therefore also among their offspring. There was no preselection during the production of these 15 generations, thus information on all the animals (including the culled animals) was used to inform selection decisions. The breeding goal consisted of a single quantitative trait that was measured in both sexes. Simulations were carried out with heritabilities of 0.5, 0.3 and 0.1, to represent breeding goal traits with high, medium and low heritabilities, respectively. Pedigree of all animals (from generations 0 to 15), genotypes of all animals in generations 13 to 15 and phenotypes of all animals in generations 11 to 15 were used in this study.

### Implementation of preselection

Preselection was implemented in generation 15 by performing several scenarios, which were combinations of three intensities of preselection and three types of preselection, across the three simulated heritabilities. An overview of these preselection scenarios is in Table 1. The three intensities of preselection investigated were no preselection (control), high preselection, and very high preselection. With no preselection, all the selection candidates (animals produced in generation 15) were kept until the subsequent genetic evaluation; thus this scenario mimicked single-stage selection. With high preselection, 10% of the male and 15% of the female selection candidates were preselected. With very high preselection, 5% of the male and 12.5% of the female selection candidates were preselected. The choice of these intensities of preselection was informed by the information that we obtained from the international breeding companies operating in the Netherlands. The three types of preselection were GPS, parent average preselection (PAPS) and random preselection (RPS). Details of the information used in each preselection type are in Table 2. Briefly, with GPS, GEBV of the selection candidates were used, which were estimated by ssGBLUP, with the phenotypes of the selection candidates excluded from the model. With PAPS, average parental GEBV of the selection candidates were used, which were estimated by ssGBLUP, with the genotypes and phenotypes of the selection candidates excluded from the model. As the name implies, RPS preselects the selection candidates randomly, and in this study, we used it to investigate the impact of reducing the number of selection candidates per se. The GEBV used in performing preselection in all scenarios of GPS and PAPS were estimated by the ssGBLUP procedure of MiXBLUP [27].

**Table 1:** Overview of the various preselection scenarios implemented

Heritability of the breeding goal trait	Type of preselection	Intensity of preselection	Scenario name
0.5	–	No^a^	No preselection with high heritability
0.5	Genomic	High^b^	High genomic preselection with high heritability
0.5	Genomic	Very high^c^	Very high genomic preselection with high heritability
0.3	–	No^a^	No preselection with medium heritability
0.3	Genomic	High^b^	High genomic preselection with medium heritability
0.3	Genomic	Very high^c^	Very high genomic preselection with medium heritability
0.1	–	No^a^	No preselection with low heritability
0.1	Genomic	High^b^	High genomic preselection with low heritability
0.1	Genomic	Very high^c^	Very high genomic preselection with low heritability
0.1	Parental average	High^b^	High parental average preselection with low heritability
0.1	Parental average	Very high^c^	Very high parental average preselection with low heritability
0.1	Random	High^b^	High random preselection with low heritability
0.1	Random	Very high^c^	Very high random preselection with low heritability

^a^No preselection: all selection candidates were kept until the subsequent genetic evaluation

^b^High preselection: 10% of the male and 15% of the female selection candidates were preselected

^c^Very high preselection: 5% of the male and 12.5% of the female selection candidates were preselected

**Table 2:** Details of the information used in the different types of preselection

Type of preselection	Preselection was based on	Information used in preselection model
Genomic	GEBV of the selection candidates^a^	Complete pedigree^b^, genotypes of all the animals in generations 13 to 15, phenotypes of all the animals in generations 11 to 14
Parent average	Average parental GEBV of the selection candidates^a^	Complete pedigree^b^, genotypes of all the animals in generations 13 and 14, phenotypes of all the animals in generations 11 to 14
Random	Random	Random numbers

^a^Selection candidates were the animals in generation 15

^b^The complete pedigree consisted of all the animals from the founder generation (generation 0) to the most recent generation (generation 15)

### Subsequent genetic evaluation

Following each preselection scenario, we performed a subsequent genetic evaluation with ssGBLUP. The subsequent evaluations included pedigree information of all the animals from generation 0 to preselected generation 15, genotypes of all the animals from generation 13 to preselected generation 15 and phenotypes of all the animals from generation 11 to preselected generation 15. This means that no information from the preculled animals was used in the subsequent evaluations. These (subsequent) evaluations provided the breeding values that were used to finally select the 100 males and 1000 females in generation 15 that become the parents of the next generation. MiXBLUP [27] was also used in these (subsequent) evaluations. Each step (simulation of the breeding program, implementation of preselection and subsequent genetic evaluations) was replicated 10 times.

### Implementation of single-step GBLUP

In order to make sure that any observed bias and loss in accuracy in our results were due to preselection, all other known possible sources of bias and loss in accuracy in ssGBLUP evaluations were accounted for. Thus, the inverse of our combined pedigree-genomic relationship matrix (${\mathbf{H}}^{ - 1}$) was as follows:

$${\mathbf{H}}^{ - 1} = {\mathbf{A}}^{ - 1} + \left[ {\begin{array}{*{20}c} 0 & 0 \\ 0 & {\left( {0.9{\mathbf{G}}_{{\mathbf{t}}} + 0.1{\mathbf{A}}_{22} } \right)^{ - 1} - {\mathbf{A}}_{22}^{ - 1} } \\ \end{array} } \right].$$

where ${\mathbf{A}}^{ - 1}$ is the inverse of pedigree relationship matrix, and ${\mathbf{A}}_{22}$ is the pedigree relationship matrix among genotyped animals. To avoid the bias that is caused by not considering inbreeding in the construction of ${\mathbf{A}}^{ - 1}$ and ${\mathbf{A}}_{22}$ [28], we considered inbreeding in both ${\mathbf{A}}^{ - 1}$ and ${\mathbf{A}}_{22}$, and the inbreeding coefficients were calculated using the algorithm of Meuwissen and Luo [29]. ${\mathbf{G}}_{{\mathbf{t}}}$ is the adjusted genomic relationship matrix that was obtained according to the *F*_ST_ method described by Powell et al. [30] and Vitezica et al. [12] and aimed at setting the average genomic inbreeding equal to the average pedigree inbreeding as follows:

$${\mathbf{G}}_{t} \, = \,\left( {1 - \overline{{f_{p} }} } \right)\,{\mathbf{G}}_{r} \, + \,2\overline{{f_{p} }} \,{\mathbf{J}}$$

where $\overline{{f_{p} }}$ is the average pedigree inbreeding coefficient across genotyped animals, ${\mathbf{G}}_{{\mathbf{r}}}$ is the raw genomic relationship matrix computed following the first method of VanRaden [31], and ${\mathbf{J}}$ is a matrix of 1s. To obtain ${\mathbf{G}}_{{\mathbf{r}}}$, we calculated allele frequencies using all the available genotypic data, and set the minor allele frequency threshold at 0.005.

The additive genetic and residual variances supplied to MiXBLUP (per heritability, per replicate) were estimated by fitting an animal model in ASReml [32]. To obtain these variances, we used the pedigree of all the animals in generations 0 to 14 and the phenotypes of all the animals in generations 11 to 14 (i.e. the available pedigree and phenotypic information at the time the selection candidates were born). The full MiXBLUP instruction file for the ssGBLUP analysis is included in Additional file 2.

### Indicators of model performance across preselection scenarios

The following indicators of model performance were estimated for each preselection scenario and compared among the scenarios.

#### (Pre)selection accuracy

Accuracy was calculated as the correlation between (G)EBV and true breeding values (TBV). After running the preselection model, preselection accuracy was calculated based on all the selection candidates, whereas after running the subsequent genetic evaluation model, the subsequent selection accuracy was computed based only on the preselected animals.

#### Bias

Bias was measured in two ways. First, the absolute bias was calculated as the difference between mean TBV and mean (G)EBV of all the preselected animals, and expressed in additive genetic standard deviation (SD) units. If there is no absolute bias, the difference is 0. A negative difference means on average (G)EBV overestimate TBV, and a positive difference means that on average (G)EBV underestimate TBV. In order to make TBV comparable to (G)EBV, we subtracted the mean TBV and the mean (G)EBV of the animals in generations 11 to 14 from the TBV and the (G)EBV of each of the preselected animals, respectively. Second, dispersion bias was measured as the regression coefficient of TBV on (G)EBV (b_TBV,(G)EBV_) of all preselected animals. If there is no dispersion bias, b_TBV,(G)EBV_ is 1. A value of b_TBV,(G)EBV_ lower than 1 means that variance of (G)EBV is inflated compared to variance of TBV, and a value of b_TBV,GEBV_ higher than 1 means that variance of (G)EBV is deflated compared to variance of TBV.

#### Realised genetic gain (RGG)

The realised genetic gain (RGG) is the difference between the average TBV of the selected individuals in two subsequent generations, provided that each of the selected animal (per sex) contributes equally to the next generation. In this study, RGG is the difference between the average TBV of the 100 males and 1000 females that were subsequently selected in generation 15 and the average TBV of the 100 males and 1000 females selected in generation 14. For each generation, we computed averages within selected males and females, separately, and then took the average of these two averages. Here, we assumed that just as in the previous generations, all the subsequently selected animals of generation 15 would have equal contributions (per sex) to the next generation. To give RGG a reference point, it was expressed in units of additive genetic SD. In reality, RGG is estimated using (G)EBV, because TBV are not known. Any bias in (G)EBV could lead to bias in estimated RGG. Thus, we calculated RGG based on (G)EBV as well. These two parameters were named true realised genetic gain (TRGG) and estimated realised genetic gain (ERGG), respectively.

## Results

Results of the genetic evaluations in which ssGBLUP was used in the subsequent evaluations are in Tables 3 and 4. The results in Table 3 are from the evaluations that were obtained with different intensities of GPS and different heritabilities. The results in Table 4 are from the evaluations that were obtained with different intensities and types of preselection, all with a heritability of 0.1.

**Table 3:** ssGBLUP performance ^a^, with different heritabilities and GPS^b^ intensities

Measure/intensity of GPS	No^c^	High^d^	Very high^e^
Heritability of 0.5			
Preselection accuracy^f^	Not applicable	0.81 (0.81–0.81)	0.81 (0.81–0.81)
Subsequent selection accuracy^g^	0.88 (0.88–0.88)	0.66 (0.64–0.68)	0.65 (0.63–0.67)
Absolute bias^h^	0.01 (0.01–0.01)	0.03 (0.01–0.05)	0.04 (0.02–0.06)
Dispersion bias^i^	1.02 (1.02–1.02)	1.06 (1.04–1.08)	1.05 (1.03–1.07)
True realized genetic gain^j^	1.53 (1.49–1.57)	1.48 (1.44–1.52)	1.45 (1.41–1.49)
Estimated realized genetic gain^k^	1.50 (1.48–1.52)	1.45 (1.43–1.47)	1.43 (1.41–1.45)
Heritability of 0.3			
Preselection accuracy^f^	Not applicable	0.78 (0.78–0.78)	0.78 (0.78–0.78)
Subsequent selection accuracy^g^	0.86 (0.86–0.86)	0.59 (0.57–0.61)	0.58 (0.56–0.60)
Absolute bias^h^	0.01 (0.01–0.01)	0.02 (0.00–0.04)	0.02 (0.00–0.04)
Dispersion bias^i^	1.01 (1.01–1.01)	1.01 (0.97–1.05)	0.98 (0.94–1.02)
True realized genetic gain^j^	1.46 (1.42–1.50)	1.39 (1.35–1.43)	1.37 (1.33–1.41)
Estimated realized genetic gain^k^	1.45 (1.41–1.49)	1.41 (1.37–1.45)	1.39 (1.35–1.43)
Heritability of 0.1			
Preselection accuracy^f^	Not applicable	0.71 (0.69–0.73)	0.71 (0.69–0.73)
Subsequent selection accuracy^g^	0.80 (0.78–0.82)	0.48 (0.44–0.52)	0.48 (0.44–0.52)
Absolute bias^h^	0.01 (0.01–0.01)	0.03 (0.01–0.05)	0.03 (0.01–0.05)
Dispersion bias^i^	1.02 (1.00–1.04)	1.01 (0.95–1.07)	1.00 (0.94–1.06)
True realized genetic gain^j^	1.38 (1.32–1.44)	1.26 (1.18–1.34)	1.24 (1.16–1.32)
Estimated realized genetic gain^k^	1.36 (1.30–1.42)	1.28 (1.22–1.34)	1.26 (1.20–1.32)

^a^Results shown only for selection candidates in the most recent generation (i.e. generation 15), and the results are means of 10 replicates (and 95% confidence intervals)

^b^Genomic preselection

^c^No preselection

^d^10% of the male and 15% of the female selection candidates were preselected

^e^5% of the males and 12.5% of the female selection candidates were preselected

^f^Correlation between true and genomic estimated breeding values of all selection candidates

^g^Correlation between true and genomic estimated breeding values of all preselected animals

^h^Difference between average true breeding value and average genomic estimated breeding value of preselected animals, expressed in additive genetic standard deviation

^i^Coefficient of the regression of true on genomic estimated breeding values of the preselected animals

^j^Difference between average true breeding value of the subsequently selected animals and average true breeding value of their parents, expressed in additive genetic standard deviation

^k^Difference between average genomic estimated breeding value of the subsequently selected animals and average genomic estimated breeding value of their parents, expressed in additive genetic standard deviation

**Table 4:** ssGBLUP performance, with different preselection types and intensities, all with a heritability of 0.1

Measure	Type and intensity of preselection						
	No preselection	High GPS	Very high GPS	High PAPS	Very high PAPS	High RPS	Very high RPS
Preselection accuracy^a^	Not applicable	0.71 (0.69–0.73)	0.71 (0.69–0.73)	0.44 (0.42–0.46)	0.44 (0.42–0.46)	0.00 (0.00–0.00)	0.00 (0.00–0.00)
Subsequent selection accuracy^b^	0.80 (0.78–0.82)	0.48 (0.44–0.52)	0.48 (0.44–0.52)	0.69 (0.67–0.71)	0.68 (0.66–0.70)	0.73 (0.71–0.75)	0.73 (0.73–0.73)
Absolute bias^c^	0.01 (−0.01–0.03)	0.03 (0.01–0.05)	0.03 (0.01–0.05)	0.03 (0.01–0.05)	0.04 (0.02–0.06)	0.05 (0.01–0.09)	0.05 (0.01–0.09)
Dispersion bias^d^	1.02 (1.00–1.04)	1.01 (0.95–1.07)	1.00 (0.94–1.06)	1.01 (0.99–1.03)	1.01 (0.97–1.05)	1.01 (0.97–1.05)	1.02 (0.98–1.06)
True realized genetic gain^e^	1.38 (1.32–1.44)	1.26 (1.18–1.34)	1.24 (1.16–1.32)	1.11 (1.03–1.19)	1.00 (0.92–1.08)	0.58 (0.54–0.62)	0.39 (0.37–0.41)
Estimated realized genetic gain^f^	1.36 (1.30–1.42)	1.28 (1.22–1.34)	1.26 (1.20–1.32)	1.12 (1.06–1.18)	1.01 (0.95–1.07)	0.58 (0.56–0.60)	0.37 (0.35–0.39)

Results shown only for the selection candidates in the most recent generation (i.e. generation 15), and the results are means over 10 replicates (and 95% confidence intervals)

Types of preselection: *GPS* genomic preselection; *PAPS* parent average preselection; *RPS* random preselection

Intensities of preselection: No preselection; high preselection - 10% of the male and 15% of the female selection candidates preselected; very high preselection - 5% of the male and 12.5% of the female selection candidates preselected

^a^Correlation between true and genomic estimated breeding values of all the selection candidates

^b^Correlation between true and genomic estimated breeding values of the preselected animals

^c^Difference between average true breeding value and average genomic estimated breeding value of preselected animals, expressed in additive genetic standard deviation

^d^Coefficient of regression of true on genomic estimated breeding values of the preselected animals

^e^Difference between average true breeding value of subsequently selected animals and average true breeding value of their parents, expressed in additive genetic standard deviation

^f^Difference between average genomic estimated breeding value of the subsequently selected animals and average genomic estimated breeding value of their parents, expressed in additive genetic standard deviation

### (Pre)selection accuracy

#### *Preselection accuracy*

Within the same heritability and type of preselection, preselection accuracy was the same for the high and very high intensities of preselection (Tables 3 and 4). GPS provided a higher preselection accuracy (0.71) than PAPS (0.44), and as expected, RPS provided a preselection accuracy equal to zero (Table 4).

#### *Subsequent selection accuracy*

For a given heritability, subsequent selection accuracy was always highest without preselection. It decreased with preselection (ranging from 0.80 to 0.48 for the scenarios with a heritability of 0.1), but within the same type of preselection, it remained similar across high and very high intensities of preselection (Tables 3 and 4). For a given heritability, subsequent selection accuracy increased from GPS to PAPS, and from PAPS to RPS (Table 4).

#### Bias

Both absolute and dispersion bias were always numerically very small, and often not statistically significant. The highest observed absolute bias was 0.05 genetic SD units, and the highest deviation of the ${\text{b}}_{{{\text{TBV}},{\text{GEBV}}}}$ from 1 (indicator of dispersion bias) was 0.06. Thus, the impacts of intensity of preselection and type of preselection on bias are considered negligible across all heritabilities.

#### Realised genetic gain

With the same heritability and type of preselection, RGG (both TRGG and ERGG) always decreased with increasing intensity of preselection (Tables 3 and 4). With the same intensity of preselection, RGG decreased from GPS to PAPS and from PAPS to RPS (Table 4), and ranged from 0.39 to 1.38 genetic SD (TRGG) and 0.37 to 1.36 genetic SD (ERGG) for the scenarios with heritability of 0.1. Irrespective of intensity of preselection, type of preselection, and heritability, ERGG was never statistically different from its corresponding TRGG (Tables 3 and 4).

## Discussion

In this study, we investigated, for different heritabilities, the impact of intensity and type of preselection on the subsequent evaluation of preselected animals in terms of selection accuracy, bias and genetic gain, using ssGBLUP with all the information from preculled animals excluded. We implemented only one stage of preselection and only one type of preselection at a time, to clearly identify the impact of each type and intensity of preselection. However, in reality, most breeding programs involve at least two stages of preselection, i.e. a first preselection of elite families using PAPS and then genotyping some members of these elite families for performing GPS. In addition, female selection candidates may not be genotyped in all cases. It is expected that, in the near future, genotyping costs will become so cheap that breeding companies will decide to genotype all their selection candidates [33]. In addition, based on our findings (i.e. that GPS hardly leads to any significant loss of genetic gain whereas PAPS does), breeding companies may become more inclined to genotype all their selection candidates so that they can perform GPS as the only type of preselection.

### Bias

We observed negligible bias in our subsequent evaluations with ssGBLUP. Patry and Ducrocq [4] have shown that PBLUP following GPS underestimates the genetic trend and decreases the accuracy of EBV of young bulls and of their daughters. Therefore, we hypothesized that our observed lack of bias was due to using ssGBLUP in the subsequent evaluations. To show this, we repeated the subsequent evaluations for our preselection scenarios with a heritability of 0.1, this time using PBLUP, with all the other parameters left unchanged. The results of the PBLUP evaluations are in Table 5. Subsequent evaluations with ssGBLUP (Table 4) resulted in higher accuracies, lower or at least similar biases, and higher realized genetic gains than the corresponding PBLUP evaluations (Table 5). Without preselection or with RPS, bias (in both absolute and dispersion forms) was absent with PBLUP, just as with ssGBLUP. Without preselection, or with an ineffective preselection such as RPS (as shown from preselection accuracies in Tables 3, 4 and 5), no preselection bias is expected. However, with GPS and PAPS, where preselection was effective (as shown from preselection accuracies in Tables 3, 4 and 5), bias was always statistically significant with PBLUP (absolute bias ranging from 0.20 to 0.50 additive genetic SD, and b_TBV,EBV_ ranging from 0.71 to 0.46), as opposed to being insignificant with ssGBLUP (absolute bias ranging from 0.03 to 0.04 additive genetic SD, and b_TBV,EBV_ always not statistically different from 1). This comparison indeed confirms that with preselection, the observed bias in subsequent genetic evaluations based on PBLUP, is removed by using ssGBLUP.

**Table 5:** PBLUP performance, with different preselection types and intensities, all with heritability of 0.1

Measure	Type and intensity of preselection						
	No preselection	High GPS	Very High GPS	High PAPS	Very high PAPS	High RPS	Very high RPS
Preselection accuracy^a^	Not applicable	0.71 (0.69–0.73)	0.71 (0.69–0.73)	0.44 (0.42–0.46)	0.44 (0.42–0.46)	0.00 (0.00–0.00)	0.00 (0.00–0.00)
Subsequent selection accuracy^b^	0.46 (0.44–0.48)	0.25 (0.21–0.29)	0.24 (0.20–0.28)	0.32 (0.28–0.36)	0.30 (0.26–0.34)	0.33 (0.31–0.35)	0.31 (0.29–0.33)
Absolute bias^c^	0.00 (−0.02-0.02)	0.42 (0.38–0.46)	0.50 (0.46–0.54)	0.20 (0.16–0.24)	0.25 (0.23–0.27)	0.00 (−0.04–0.04)	0.00 (−0.04–0.04)
Dispersion bias^d^	0.98 (0.94–1.02)	0.47 (0.41–0.53)	0.46 (0.38–0.54)	0.71 (0.65–0.77)	0.67 (0.61–0.73)	0.99 (0.91–1.07)	0.98 (0.90–1.06)
True realized genetic gain^e^	0.79 (0.73–0.85)	1.12 (1.06–1.18)	1.16 (1.10–1.22)	0.84 (0.76–0.92)	0.82 (0.74–0.90)	0.26 (0.24–0.28)	0.15 (0.13–0.17)
Estimated realized genetic gain^f^	0.81 (0.75–0.87)	0.47 (0.43–0.51)	0.39 (0.35–0.43)	0.54 (0.50–0.58)	0.46 (0.42–0.50)	0.27 (0.25–0.29)	0.17 (0.15–0.19)

Results shown only for the selection candidates in the most recent generation (i.e. generation 15), and the results are means over 10 replicates (and 95% confidence intervals)

Types of preselection: *GPS* genomic preselection; *PAPS* parent average preselection; *RPS* random preselection

Intensities of preselection: no preselection; high preselection–10% of the male and 15% of the female selection candidates preselected; very high preselection–5% of the male and 12.5% of the female selection candidates preselected

^a^Correlation between true and genomic estimated breeding values of all the selection candidates

^b^Correlation between true and estimated breeding values of the preselected animals

^c^Difference between average true breeding value and average estimated breeding value of preselected animals, expressed in additive genetic standard deviation

^d^Coefficient of regression of true on estimated breeding values of the preselected animals

^e^Difference between average true breeding value of subsequently selected animals and average true breeding value of their parents, expressed in additive genetic standard deviation

^f^Difference between average estimated breeding value of the subsequently selected animals and average estimated breeding value of their parents, expressed in additive genetic standard deviation

### Subsequent selection accuracy

Subsequent selection accuracy decreased with preselection, and this is in line with the findings reported by Patry and Ducrocq [4] in PBLUP evaluations following GPS in dairy cattle breeding schemes. It is important to note that without preselection, the subsequent selection accuracy was calculated across many more animals (16,000) compared to the 2000 and 1400 animals, respectively, used to for high and very high preselection scenarios. Even when the subsequent selection accuracy in the scenario without preselection was calculated using only these 2000 or 1400 preselected animals, it was still higher than in the scenarios with preselection [see Additional file 3]. The explanation for this result is that each selection candidate had, on average, more full and half sibs at the subsequent genetic evaluation without preselection than in the high and with very high preselection scenarios, and the phenotypes of these additional full and half sibs added to the accuracy of the scenario without preselection. With different types of preselection, contrary to the trend that we observed with preselection accuracy, the subsequent selection accuracy increased from GPS to PAPS, and from PAPS to RPS, because the more accurate the preselection was, the lower the additive genetic variance left in the preselected animals [34, 35], which in turn reduced selection accuracy [35].

### Realised genetic gain (RGG)

We observed a decrease in RGG (both TRGG and ERGG) as intensity of preselection increased. The reason for this is that as intensity of preselection increased, more of the best animals (in terms of TBV) were lost during preselection, since preselection was never 100% accurate. Other studies have reported a similar trend, i.e. a reduction in genetic gain with an increasing intensity of preselection, and offered similar explanations (e.g. [2, 36–38]). With different types of preselection, we observed that RGG depended more on preselection accuracy than on subsequent selection accuracy, and therefore RGG had a trend that was more similar to the trend of preselection accuracy than to that of subsequent selection accuracy (Table 4). The reason is that among preselection types, variation in preselection accuracy was larger than that in subsequent selection accuracy (Table 4), due to different sources of information used in each preselection type (Table 2). In the subsequent genetic evaluations, irrespective of the type of preselection, the model used all three sources of information, i.e. pedigree, genotypes and phenotypes of the preselected candidates. This explains why RGG was always higher with GPS, than with PAPS, and why the lowest genetic gain was recorded with RPS. Schrooten et al. [2] also reported a larger impact of preselection accuracy than of subsequent selection accuracy on genetic gain in dairy cattle breeding schemes.

### GPS and RGG

The decrease in RGG from no preselection to high and very high GPS scenarios was always small. Specifically, TRGG and ERGG decreased by 3.3 to 8.7% and 2.8 to 5.9%, respectively, from no preselection to high GPS, depending on heritability (Table 3). With the very high intensity of preselection, the number of females required to produce the next generation in this study (1000 females) was already reached at the preselection stage, thus there was no selection in females at the subsequent selection stage. TRGG and ERGG decreased, by 5.2 to 10.1% and 4.1 to 7.4%, respectively, from no preselection to very high GPS, depending on heritability (Table 3). These results show that, with ssGBLUP evaluations following GPS, it is possible to achieve a level of genetic gain that is similar to that achieved without preselection. This is especially important for traits that are expensive to measure (e.g. feed intake of individual broiler chickens), and traits for which phenotypes can only be measured at advanced stages of life (e.g. litter size in pigs). For such traits, GPS enables saving on the cost of phenotyping the preculled animals, and on the cost of raising the preculled animals in the expensive nucleus environments of breeding programs.

## Conclusions

Using ssGBLUP in subsequent genetic evaluations prevents preselection bias, irrespective of intensity and type of preselection, and heritability. With GPS, in addition to reducing the phenotyping effort considerably, the use of ssGBLUP in subsequent genetic evaluations realizes only a slightly lower genetic gain than that realized without preselection. This is especially the case for traits that are expensive to measure (e.g. feed intake of individual broiler chickens), and traits for which phenotypes can only be measured at advanced stages of life (e.g. litter size in pigs).

## Supplementary information

**Additional file 1:** QMSim parameter file. The QMSim parameter file used to simulate the data used in this study.

**Additional file 2**: MiXBLUP instruction file. The MiXBLUP instruction file used for data analysis in this study.

**Additional file 3**. Accuracy of the no preselection (control) scenario in subsequent ssGBLUP evaluations, calculated across different animals. Accuracies of the no preselection (control) scenario in subsequent ssGBLUP evaluations, calculated across the different sets of selection candidates preselected by each preselection scenario.

## Data Availability

The codes used in generating the data used in this study are attached to this article as Additional file 1.
