# Supplementary material for: Investigating the impact of preselection on subsequent single-step genomic BLUP evaluation of preselected animals
Source: Genet Sel Evol. 2020 Jul 29;52:42. doi: 10.1186/s12711-020-00562-6 (PMC7392691; doi:10.1186/s12711-020-00562-6)
Supplement: Supplementary file 1 — Additional file 1: QMSim parameter file. The QMSim parameter file used to simulate the data used in this study. [file 12711_2020_562_MOESM1_ESM.docx]

**Additional file 1** **QMSim parameter file**

/*** Global parameters ***/

title = "title";

seed = "seed_main.prv"; // use the file "seed_main.prv" as the seed file

nrep = 10; // replicate 10 times

h2 = 0.3; // total h2

qtlh2 = 0.27; // proportion of h2 explained by QTL

phvar = 100; // total phenotypic variance

/*** Historical polulation ***/

begin_hp;

hg_size = 5000 [0] 50 [2997] 5000 [3000]; // start with 5000 animals at generation 0, keep reducing the population size until it reaches 50 animals at generation 2997, and then start raising it until it reaches 5000 animals again at generation 3000. Maintain equal sex ratio across all the generations.

/*** Recent polulation ***/

begin_pop = "rp";

begin_founder;

male [n = 100, pop = "hp"];

female [n = 1000, pop = "hp"]; // select the founder population from the last generation of historical population. Select 100 males and 1000 females randomly to form the founder population. Also, select 100 males and 1000 females per generation to produce the next generation, based on the selection and mating criteria below:

end_founder;

ng = 15; // simulate 15 generations of recent population

ls= 16; // litter size is 16 offspring per dam

pmp = 0.5 /fix_litter; // maintain equal sex ratio per litter (so each litter has 8 male and 8 female offspring)

md = minf; // mating design is to minimize inbreeding

sd = ebv /h; // selection is based on highest EBV

ebv_est = blup; // estimate EBV using pedigree BLUP

begin_popoutput;

data; // save all individual’s data except their genotypes

stat; // save brief statistics on simulated data

allele_freq /mafbin 50; // save allele frequencies, with 50 bins for minor allele frequency distribution

genotype /gen 13 14 15; // save genotype data for generations 13, 14, and 15 only

end_popoutput;

end_pop;

/*** Genome section ***/

begin_genome;

begin_chr = 30; // simulate 30 chromosomes

chrlen = 100; // each should be 100cM in length

nmloci = 5000; // simulate 5000 markers per chromosome. This will ensure that at the end of the historical population, we have about 2000 markers per chromosome with MAF $\geq$ 0.005, as about 60% of the markers end up with MAF < 0.005.

mpos = even /start 0.5 /end 99.5; // place the markers evenly between 0.5 cM and 99.5 cM

nma = all 2; // all markers should have two alleles each at the beginning of the historical population

maf = eql; // at the 1st generation of historical population, all marker alleles should have equal frequency (0.5)

nqloci = 250; // simulate 250 QTL per chromosome. This will ensure that at the end of the historical population, we have about 100 QTL per chromosome with MAF >= 0.005, as about 60% of the QTL end up with a MAF < 0.005.

qpos = even /start 1 /end 99; // place the QTL between 1st and the 99th cM

nqa = all 2; // all QTL should have two alleles each at the beginning of the historical population

qaf = eql; // at the 1st generation of historical population, all QTL alleles should have equal frequency (0.5)

qae = rndg 0.4; // QTL allele effects should be randomly drawn from a gamma distribution with shape parameter of 0.4

end_chr;

select_seg_loci /maft 0.005; // at the end of the historical population, consider only markers and QTL with MAF $\geq$ 0.005 in producing the recent population

mmutr = 2.5e-5 /recurrent; // marker mutation rate, and the mutation should be recurrent. This means that mutation is only possible among existing alleles and no new alleles are formed.

qmutr = 2.5e-5 /recurrent; // QTL mutation rate, and the mutation should be recurrent

end_genome;

/*** General output ***/

begin_output;

linkage_map; // save linkage map

allele_effect; // save marker and QTL allele substitution effects

hp_stat; // save statistics of the historical population

end_output;
