# Supplementary material for: Investigating the impact of preselection on subsequent single-step genomic BLUP evaluation of preselected animals
Source: Genet Sel Evol. 2020 Jul 29;52:42. doi: 10.1186/s12711-020-00562-6 (PMC7392691; doi:10.1186/s12711-020-00562-6)
Supplement: Supplementary file 2 — Additional file 2: MiXBLUP instruction file. The MiXBLUP instruction file used for data analysis in this study. [file 12711_2020_562_MOESM2_ESM.docx]

**Additional file 2** **MiXBLUP instruction file**

***mixblup_ssGBLUP # title of the analysis***

***# Observations & systematic effects***

DATAFILE pheno # name of the file with phenotypic data

animal I # the ID of the animals is in the first column, and it is in numeric form

sire I # the sires of the animals are in the second column, and they are in numeric form

dam I # the sires of the animals are in the second column, and they are in numeric form

sex A # sex of the animal, and it is in alphanumeric form

phen T # the trait of interest

***# Genetic similarity among individuals***

ERMFILE geno !CONSTRUCT SSmat # the genotype file; make a new weighted $\mathbf{G}^{-1}$ in the MiXBLUP parser from the genotype file

animal I # the ID of the animals is in the first column, and it is in numeric form

!METHOD VanRaden # use the first method of VanRaden in making **G**

!DENSE #markers provided arevin dense format

!MAF 0.005 # MAF threshhold

!STORE_GINV # store $\mathbf{G}^{-1}$ in the right format to be re-used by calc_grm.

!NUMPROC 5 # the number of threads to be used by calc_grm.

!LAMBDA 1 # weighing factor for $\mathbf{G}^{-1}$ in making $\mathbf{H}^{-1}$

!ALPHA 0.9 # weighing factor for $\mathbf{G}$ in making $\mathbf{G}^{-1}$

!BETA 0.1 # weighing factor for $\mathbf{A}_{22}^{\boldsymbol{-}1}$ in making $\mathbf{G}^{-1}$

!OMEGA 1 # weighing factor for $\mathbf{A}_{22}^{\boldsymbol{-}1}$in making $\mathbf{H}^{-1}$

!SINGLESTEP # MiXBLUP kernel should calculate the $\mathbf{H}^{-1}$ from a $\mathbf{G}^{-1}$, the pedigree file and a file with inbreeding coefficients

PEDFILE pedi !CALCINBR # pedigree file, calculate inbreeding coefficients from it

animal I # the ID of the animals is in the first column, and it is in numeric form

sire I # the sires of the animals are in the second column, and they are in numeric form

dam I # the sires of the animals are in the second column, and they are in numeric form

***# Components of variance and covariance among traits***

PARFILE VCfile # the file containing additive genetic and residual variances

***# Statistical models***

MODEL

phen ~ mu !RANDOM G(animal) # the phenotype of interest is a function of overall mean and genomic differences among the animals

***# Control of analysis and output***

SOLVING # use the below information to control the process and the output of the analysis.

!STOPCRIT 1.0E-06 # convergence criterion

!NOPEEK # do not store preliminary results

TMPDIR /destination # working directory
