# Supplementary material for: Investigating the impact of preselection on subsequent single-step genomic BLUP evaluation of preselected animals
Source: Genet Sel Evol. 2020 Jul 29;52:42. doi: 10.1186/s12711-020-00562-6 (PMC7392691; doi:10.1186/s12711-020-00562-6)
Supplement: Supplementary file 3 — Additional file 3. Accuracy of the no preselection (control) scenario in subsequent ssGBLUP evaluations, calculated across different animals. Accuracies of the no preselection (control) scenario in subsequent ssGBLUP evaluations, calculated across the different sets of selection candidates preselected by each preselection scenario. [file 12711_2020_562_MOESM3_ESM.docx]

**Additional file 3 Accuracy^a^ of the no preselection (control) scenario in subsequent ssGBLUP evaluations, calculated across different animals^b^**

| **Calculated across** | **Accuracy^a^** |
| --- | --- |
| all the 16,000 selection candidates | 0.80 (0.78-0.82) |
| the 2000 selection candidates preselected under high GPS scenario | 0.62 (0.60-0.64) |
| the 1400 selection candidates preselected under very high GPS scenario | 0.62 (0.60-0.64) |
| the 2000 selection candidates preselected under high PAPS scenario | 0.75 (0.73-0.77) |
| the 1400 selection candidates preselected under very high PAPS scenario | 0.75 (0.75-0.75) |
| the 2000 selection candidates preselected under high RPS scenario | 0.80 (0.78-0.82) |
| the 1400 selection candidates preselected under very high RPS scenario | 0.80 (0.80-0.80) |

^a^Correlation between true and genomic estimated breeding values of all the preselected candidates, and results are means of 10 replicates (and 95% confidence intervals). ^b^The different animals are the animals preselected under the different types and intensities of preselection. The types of preselection are GPS - genomic preselection, PAPS - parent average preselection and RPS – random preselection. The different intensities of preselection are no preselection, high preselection - 10% of the male and 15% of the female selection candidates preselected, and very high preselection - 5% of the male and 12.5% of the female selection candidates preselected.
